# Comparison of community structures of *Candidatus* Methylomirabilis oxyfera-like bacteria of NC10 phylum in different freshwater habitats

**DOI:** 10.1038/srep25647

**Published:** 2016-05-09

**Authors:** Li-dong Shen, Hong-sheng Wu, Zhi-qiu Gao, Xu Liu, Ji Li

**Affiliations:** 1 Collaborative Innovation Center on Forecast and Evaluation of Meteorological Disasters, Jiangsu Key Laboratory of Agricultural Meteorology, College of Applied Meteorology, Nanjing University of Information Science and Technology, Nanjing, 210044, China; 2 Department of Agricultural Resource and Environment, College of Applied Meteorology, Nanjing University of Information Science and Technology, Nanjing 210044, China; 3 State Key Laboratory of Atmospheric Boundary Layer Physics and Atmospheric Chemistry, Institute of Atmospheric Physics, Chinese Academy of Science, Beijing, China; 4 College of Geophysics and Remote Sensing, Nanjing University of Information Science and Technology, Nanjing 210044, China

## Abstract

Methane oxidation coupled to nitrite reduction is mediated by ‘*Candidatus* Methylomirabilis oxyfera’ (*M. oxyfera*), which belongs to the NC10 phylum. In this study, the community composition and diversity of *M. oxyfera*-like bacteria of NC10 phylum were examined and compared in four different freshwater habitats, including reservoir sediments (RS), pond sediments (PS), wetland sediments (WS) and paddy soils (PAS), by using Illumina-based 16S rRNA gene sequencing. The recovered NC10-related sequences accounted for 0.4–2.5% of the 16S rRNA pool in the examined habitats, and the highest percentage was found in WS. The diversity of NC10 bacteria were the highest in RS, medium in WS, and lowest in PS and PAS. The observed number of OTUs (operational taxonomic unit; at 3% cut-off) were 97, 46, 61 and 40, respectively, in RS, PS, WS and PAS. A heterogeneous distribution of NC10 bacterial communities was observed in the examined habitats, though group B members were the dominant bacteria in each habitat. The copy numbers of NC10 bacterial 16S rRNA genes ranged between 5.8 × 10^6^ and 3.2 × 10^7^ copies g^−1^ sediment/soil in the examined habitats. These results are helpful for a systematic understanding of NC10 bacterial communities in different types of freshwater habitats.

Methane (CH_4_) is the most important greenhouse gas after carbon dioxide, and responsible for ~20% of the current greenhouse effect^1^. Freshwater habitats like natural wetlands are an important source of atmospheric methane^1^,^2^. In the freshwater habitats, aerobic methane oxidation using oxygen as electron acceptor is the most important biological sink of methane, but the role of alternative electron donors is not well known^3^. Recently, evidence showed that methane oxidation coupled to nitrite reduction may also play an important role in reducing methane emissions from different freshwater habitats^4^,^5^,^6^,^7^,^8^,^9^,^10^.

Methane oxidation coupled to nitrite reduction was first demonstrated in an enrichment culture from freshwater sediments^11^,^12^,^13^. The organism responsible for this reaction has been identified as *Candidatus* Methylomirabilis oxyfera (*M. oxyfera*), which belongs to the NC10 phylum^14^. This phylum was proposed by Rappe and Giovannoni^15^ based on 16S rRNA gene sequences recovered from flooded caves, and has no members in pure culture. Based on 16S rRNA gene or functional gene clone libraries using specific primers, the community composition and diversity of NC10 bacteria in different freshwater habitats have previously been described separately, primarily including lakes^4^,^6^,^16^, rivers^17^, wetlands^7^,^9^,^10^,^18^ and paddy fields^8^,^19^,^20^. Given the potential role of NC10 bacteria in reducing methane emissions^21^,^22^, it is therefore imperative to examine and compare their community composition and diversity in different freshwater habitats to determine whether these habitats can influence their community structures.

The Illumina MiSeq platform provides researchers with a scalable, high-throughput and streamlined sequencing platform to survey microbial communities from environmental samples^23^. This technique has been widely used to study microbial communities in various freshwater habitats, and have changed our understanding of microbial diversity in the environment^24^,^25^,^26^. The Illumina-based 16S rRNA gene sequencing has high depth coverage of microbial diversity and can detect both abundant and rare microbes^25^,^27^,^28^, and thus is a more powerful method than the clone libraries for evaluation of microbial communities. However, until now, only Lu *et al*.^29^ and Shen *et al*.^30^ have applied Illumina-based 16S rRNA gene sequencing technology to examine the community structures of NC10 bacteria, and the latter showed an unexpected high diversity of NC10 bacteria at species level in agricultural soils. To the best of our knowledge, no study has used this technique to compare the community composition and diversity of NC10 bacteria between different natural habitats. Knowledge from comparisons of NC10 bacterial community structures in different freshwater habitats may identify habitat-specific adaptations in their physiology and evolution. It can also reveal whether certain NC10 bacterial taxa tend to be detected only in one habitat or co-occur in different habitats, which has implications for their biogeography and ecology.

In the present study, an improved dual-indexing amplification and sequencing approach developed by Fadrosh *et al*.^23^ was used to assess the community composition and diversity of NC10 bacterial communities in different freshwater habitats. Four different freshwater habitats were selected in this study, including the reservoir sediments, pond sediments, wetland sediments and paddy soils. We hypothesized that these habitats harbored unique NC10 bacterial assemblages.

## Materials and Methods

### Site description and sample collection

Different freshwater sediments/soils were selected in the current study, including the reservoir sediments (RS), pond sediments (PS), wetland sediments (WS) and paddy soils (PAS). These sediments were permanently or semi-permanently submerged by water, which can create anoxic conditions for the NC10 bacteria. Three RS samples (RS1, RS2 and RS3) were collected from the Jiulonghu Reservoir, which is located in Zhejiang Province, China. The upper 15 cm sediments were collected from this reservoir using box-cores in July 2015. Four PS samples (PS1, PS2, PS3 and PS4) were collected from four aquaculture ponds that are located in Jiangsu Province, China. The upper 15 cm sediments were collected from these ponds using box-cores in November 2014 as previously described^31^. Four sediment samples (WS1, WS2, WS3 and WS4) were collected from a natural freshwater wetland, the Baguazhou Wetland, located in Jiangsu Province, China. The upper 15 sediments were collected from this wetland system using a stainless steel ring sampler in January 2015 as previously described^32^. Three soil samples were collected from a paddy field that subject to long-term fertilization in Jiangsu Province. The soil layers of 0–10 cm (PAS10), 20–30 cm (PAS30) and 40–50 cm (PAS50) were collected from the paddy field using a stainless steel ring sampler in June 2015. All the collected sediment/soil samples were transferred immediately to sterile plastic bags, and transported to the laboratory on ice within 12 h. The collected samples were subsequently divided into two parts. The first part was stored at 4 °C for physicochemical analysis, and the other part was stored at −20 °C for later molecular analysis.

### Chemical analysis

The sediment/soil pH was determined after mixing the sediment/soil with deionized water at a ratio (sediment/soil: water) of 1:2.5. Sediment/soil ammonium and nitrate were extracted from the sediment/soil using 2 M KCl as previously described^33^, and the extracted ammonium and nitrate were determined according to the reported studies^34^,^35^. The sediment/soil organic carbon content was determined by the K_2_Cr_2_O_7_ oxidation method^36^.

### DNA isolation

Sediment/soil DNA was extracted using a Power Soil DNA kit (Mo Bio Laboratories, Carlsbad, California, USA) according to the manufacturer’s instructions. Approximately 0.25 g homogenized sediment/soil was used for DNA isolation. The quality of the extracted DNA was evaluated on 1% agarose gel, and the concentration of DNA was measured with a NanoDrop spectrophotometer (ND-1000; Isogen Life Science, the Netherlands).

### Illumina-based 16S rRNA gene sequencing

High-throughput sequencing of bacterial 16S rRNA genes was performed on the Illumina MiSeq platform (Hangzhou Guhe Information and Technology Co., Ltd., Zhejiang, China). Briefly, the V3-V4 region of bacterial 16S rRNA genes was amplified by the primer 319f (5′-ACTCCTACGGGAGGCAGCAG-3′) and primer 806r (5′-GGACTACHVGGGTWTCTAAT-3′) as previously described^23^,^30^. Briefly, PCRs were performed in a 30 μl mixture containing 0.5 μl Dimethyl sulfoxide, 1.0 μl forward primer (10 mM), 1.0 μl reverse primer (10 mM), 5.0 μl DNA template, and 15.0 μl Phusion High-Fidelity PCR Master Mix with HF Buffer (NEB). The thermal program was as follows^23^,^30^: 98 °C for 30 s; 30 cycles of 98 °C for 15 s, 58 °C for 15 s, and 72 °C for 15 s; and a final extension at 72 °C for 1 min. PCR products were purified using an agarose gel DNA purification kit (Qiagen, Chatsworth, California, USA) and then were subjected to Illumina MiSeq sequencing (2 × 300 bp). Analysis of Mi-Seq sequencing data was conducted using Quantitative Insights Into Microbial Ecology (QIIME, www.qiime.org) according to Shen *et al*.^30^,^31^. High-quality NC10 phylum bacterial sequences were further confirmed by aligning these sequences with the reported NC10 sequences deposited in GenBank using the BLAST search engine^30^. Representative sequences for each operational taxonomic unit (OTU) as defined by 97% sequence identity were obtained for further analysis. The coverage of NC10 bacteria in each sample was calculated as *C* = [1 − (*n*_1_*/N*)] × 100, where *n*_1_ is the number of unique OTUs and *N* is the total number of sequences in the sample.

### Phylogenetic analysis

Phylogenetic analysis of the recovered NC10-related 16S rRNA gene sequences was performed by Mega 5 software using the neighbour-joining method^37^, and the evolutionary distances were computed using the Maximum Composite Likelihood method. The robustness of the tree topology was tested with a bootstrap analysis (1000 replicates), and bootstrap values >50 (500 replicates) are shown at the branches.

### Quantitative PCR

The primers qp1f (5′-GGGCTTGACATCCCACGAACCTG-3′) and qp1r (5′-CGCCTTCCTCCAGCTTGACGC-3′) targeting the 16S rRNA genes of NC10 bacteria were used to determine the abundance of these bacteria as previously described^12^. The primers 341f (5′-CCTACGGGAGGCAGCAG-3′) and 518r (5′-ATTACCGCGGCTGCTGG-3′) with coverage of most bacterial 16S rRNA genes were applied to determine the abundance of total bacteria according to Muyzer *et al*.^38^. The standard curves were constructed from a series of 10-fold dilutions of a known copy number of plasmid DNA (Fig. S1). Negative controls, in which the DNA template was replaced by nuclease-free water, were also performed. Triplicate qPCR analyses were performed for each sample.

### Statistical analysis

The geographical distribution of NC10 bacterial communities and their potential correlations with environmental factors were determined using principal components analysis (PCA) and canonical correspondence analysis (CCA), respectively, using the CANOCO software^39^. Venn diagrams were built using Venny 2.0 (http://bioinfogp.cnb.csic.es/tools/venny/index.html). Pearson moment correlation analysis (significance level *α* = 0.05) was used to test for potential correlations between the NC10 bacterial diversity, abundance and different environmental factors, using the SPSS 18.0 software (SPSS, Chicago, Illinois, USA).

## Results

### Basic physicochemical properties of different freshwater habitats

The basic physicochemical properties of the examined freshwater habitats are shown in Table S1. The sediment/soil pH varied from 6.9 to 7.3, 7.2 to 7.6, 6.2 to 7.1 and 6.7 to 7.1, respectively, in the examined RS, PS, WS and PAS. Different inorganic nitrogen contents were observed in the examined habitats. The PS showed the highest contents of 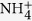-N and 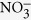-N, ranging from 52.5 to 174.6 and 15.3 to 49.2 mg kg^−1^, respectively, while the RS showed the lowest contents of 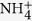-N and 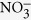-N, ranging from 3.2 to 5.3 and 0.8 to 1.4 mg kg^−1^, respectively. The 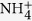-N and 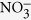-N contents in the examined WS varied from 20.8 to 43.1 and 15.6 to 29.1 mg kg^−1^, respectively, and the 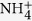-N and 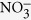-N contents in the examined PAS varied from 36.9 to 56.9 and 3.3 to 17.8 mg kg^−1^, respectively. The 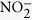-N contents were very low in the examined habitats, ranging from 0.1 to 0.2, 0.2 to 0.5, 0.1 to 0.2 and 0.1 to 0.4 mg kg^−1^, respectively, in RS, PS, WS and PAS. The organic carbon (OrgC) contents ranged from 12.5 to 13.1, 25.6 to 30.4, 10.5 to 14.6 and 13.1 to 15.2 g kg^−1^, respectively, in RS, PS, WS and PAS.

### Illumina-based 16S rRNA gene sequencing

Illumina-based 16S rRNA gene sequencing was applied to determine the presence of NC10 bacteria in the examined freshwater habitats as previously described^30^. The dominant bacterial phyla (at least 1% in average in each habitat including the candidate phylum) in RS were Proteobacteria (35.3–38.0%), Chloroflexi (14.7–17.7%), Acidobacteria (13.7–14.5%), Bacteroidetes (4.0–6.6%), Nitrospirae (1.9–4.3%), Chlorobi (2.2–2.9%), Firmicutes (2.0–2.5%), OP8 (1.2–2.5%), WS3 (1.2–2.3%), Actinobacteria (1.7–2.2%), NC10 (1.0–1.5%), Verrucomicrobia (1.3–2.0%). The NC10 was the eleventh dominant phylum in the examined RS. The dominant phyla in PS were Proteobacteria (48.0–90.2%), Bacteroidetes (2.2–29.0%), Chloroflexi (1.7–18.1%), Firmicutes (1.3–11.0%), Acidobacteria (0.9–5.8%), Nitrospirae (1.0–5.7%), OP8 (0.1–4.6%). The NC10 phylum accounted for 0.1–0.7% of the 16S rRNA pool in the examined PS, which was the eleventh dominant phylum. For the WS, the dominant phyla were Acidobacteria (21.8–42.1%), Proteobacteria (25.7–35.0%), Chloroflexi (8.6–11.5%), Nitrospirae (3.9–7.7%), Bacteroidetes (1.4–11.0%), WS3 (2.5–5.0%), NC10 (1.6–4.5%), Actinobacteria (1.6–2.7%), Gemmatimonadetes (1.7–3.2%), Planctomycetes (0.8–2.5%), Firmicutes (0.7–2.9%). The NC10 was the seventh dominant phylum in the examined WS. The dominant phyla in PAS were Proteobacteria (22.9–55.2%), Acidobacteria (5.4–32.6%), Actinobacteria (9.4–12.3%), Chloroflexi (6.2–18.9%), Nitrospirae (3.6–8.1%), GAL15 (0.4–6.9%), Gemmatimonadetes (0.5–4.9%), TM7 (0.4–2.0%), Bacteroidetes (0.5–1.6%), NC10 (0.1–2.0%). The NC10 was the tenth dominant phylum in the examined PAS. The percentage of NC10 bacteria in each sample is shown in Table S2. In addition, previous studies have indicated that anaerobic ammonium-oxidizing (anammox) bacteria (belonging to the Planctomycetes phylum), which also use nitrite as electron acceptor, could compete for nitrite with NC10 bacteria in wetland sediments^10^. It was found that Planctomycetes phylum reads accounted for a smaller part of the 16S rRNA pool, ranging from 0.1–2.5% in the examined samples (Table S2). Anammox bacteria only accounted for 0.012–0.112% of the 16S rRNA pool (Table S2).

### Diversity of NC10 phylum bacteria

The coverage values of the NC10 sequences ranged from 90.3% to 95.8%, 91.5% to 99.7%, 98.5% to 99.3% and 92.9% to 99.0%, respectively, in the examined RS, PS, WA and PAS (Table 1), indicating that the 16S rRNA gene sequences of NC10 bacteria were sufficiently represented by Illumina-based 16S rRNA gene sequencing. Different levels of diversity of NC10 bacterial 16S rRNA genes were observed in the examined habitats (Table 1). The highest diversity of NC10 bacterial 16S rRNA genes was observed in RS, with a total of 97 OTUs (44–75 OTUs in each sample) being detected. A lower diversity of NC10 bacterial 16S rRNA genes was observed in PS and PAS, with a total of 46 (19–27 OTUs in each sample) and 40 OTUs (11–33 OTUs in each sample) being detected, respectively. The WS showed intermediate level of diversity of NC10 bacterial 16S rRNA genes, with a total of 61 (27–44 OTUs in each sample) OTUs being detected. The variation of number of OUTs observed in different habitats was consistent with the variation of Chao1 estimator. As seen from the Shannon index, the diversity of NC10 bacteria were the highest in RS, medium in PS and PAS and lowest in WS (Table 1).

### Community composition of NC10 phylum bacteria

**Figure 1:**
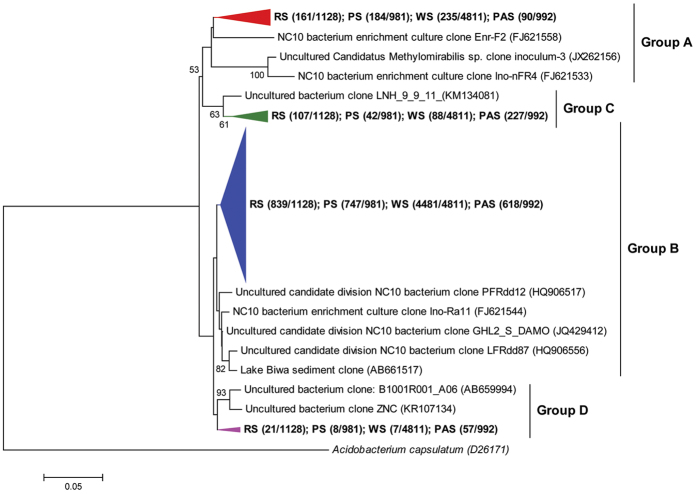
Neighbour-joining phylogenetic tree showing the phylogenetic affiliations of the 16S rRNA gene sequences of NC10 phylum bacteria recovered from the examined freshwater habitats. Bootstrap values were 1,000 replicates, and the scale bar represents 5% sequence divergence. Bootstrap values >50 (500 replicates) are shown at the branches.

Phylogenetic analysis showed that the recovered NC10 sequences could be mainly assigned to four different groups of NC10 bacteria (Fig. 1), including group A, group B, group C and group D, according to Ettwig *et al*.^13^. Sequences of group A showed 94.2–98.7% identity to the 16S rRNA gene of *M. oxyfera*, and were most closely related to the sequences recovered from *M. oxyfera* enrichment culture^12^,^13^ with 97–99% identity. Sequences of group B showed 89.0–93.5% identity to the 16S rRNA gene of *M. oxyfera*, and were most closely related to the clones obtained from the inocula of *M. oxyfera* enrichment culture^13^ and lake sediments^4^,^16^,^40^ with 96–99% identity. Sequences of group C and group D only showed 88.1–88.7% and 86.0–87.8% identities, respectively, to the 16S rRNA gene of *M. oxyfera*. The most closely related sequences of these two groups were the sequences recovered from lake water columns^41^ and paddy soils^42^, with 94–98% identity. The group B members were the dominant NC10 bacteria in each examined habitat, which were responsible for 74.4% (66.8–78.3% in each sample), 76.3% (48.9–83.5% in each sample), 91.6% (90.1–94.6% in each sample) and 62.3% (17.9–64.1% in each sample) of the total NC10-related sequences, respectively, in RS, PS, WS and PAS (Fig. S2). The group A members, which were proved to have the capability to catalyze AMO coupled to nitrite reduction, accounted for a minor portion of the NC10 bacteria. The proportions of group A members of NC10 bacteria were 14.3% (12.0–19.4% in each sample), 16.2% (12.6–45.7% in each sample), 5.6% (3.6–5.9% in each sample) and 9.1% (0–11.2% in each sample), respectively, in RS, PS, WS and PAS.

### Spatial distribution of NC10 phylum bacterial assemblages and their relationships with environmental factors

**Figure 2:**
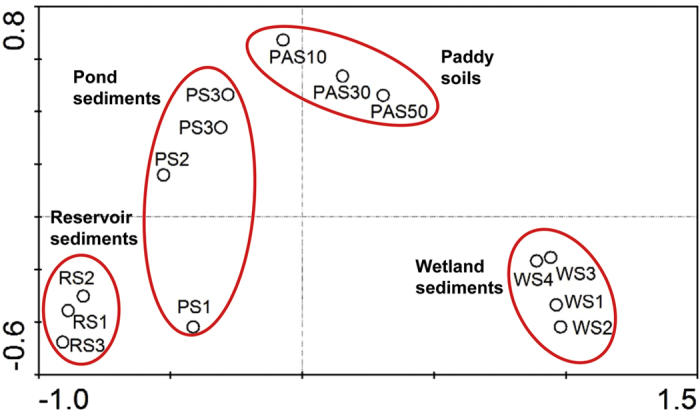
PCA ordination diagram of the NC10 phylum bacterial assemblages calculated with 16S rRNA gene sequences recovered from the examined freshwater habitats.

**Figure 3:**
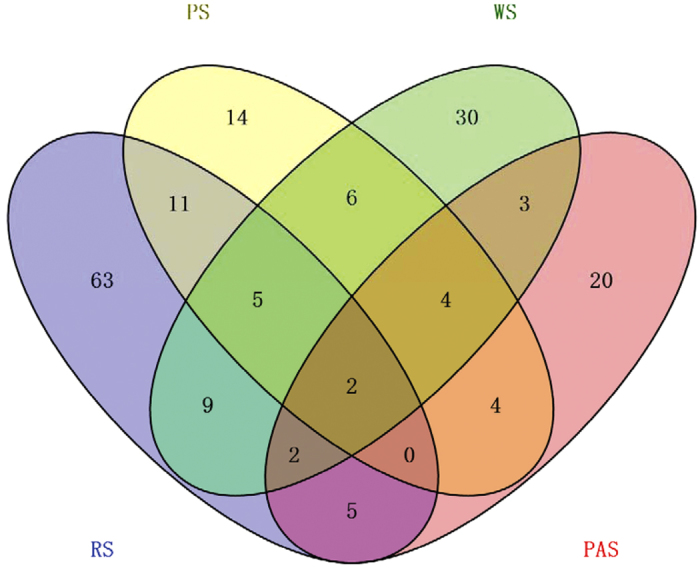
A Venn Diagram showing the unique and sharing OTUs in each examined freshwater habitat.

**Figure 4:**
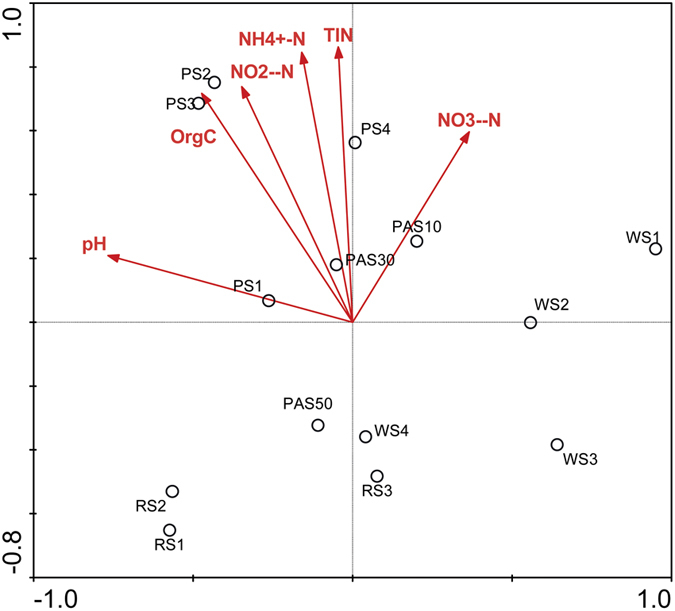
CCA ordination plots for the first dimensions to show the correlations between NC10 phylum bacterial community structures and environmental factors using the 16S rRNA gene sequences recovered from the examined freshwater habitats. The correlations between the environmental factors and CCA axes are represented by the length and angle of the arrows.

Based on PCA using the recovered 16S rRNA gene sequences, the community structures of NC10 bacterial assemblages appeared to differ substantially among the four different habitats. The NC10 bacterial communities fell into four different groups: RS group, PS group, WS group and PAS group (Fig. 2). It was found that the WS was more isolated from the remaining groups. Furthermore, a Venn diagram showed that over half (64.9%; 63 of 97 OTUs) of the OTUs in RS are unique and are not detected in any other habitats (Fig. 3). For the remaining habitats, approximately 30.4% (14 of 46 OTUs), 49.2% (30 of 61 OTUs) and 50% (20 of 40 OTUs) OTUs are unique in PS, WS and PAS, respectively. To find the potential correlations between the distribution of NC10 bacterial assemblages and the environmental factors of the examined habitats, CCA test was conducted based on the recovered NC10 bacterial 16S rRNA gene sequences and the measured physicochemical parameters. It was found that the sediment/soil ammonium content and total inorganic nitrogen (TIN) content appeared to be the most significant of the investigated environmental factors with respect to the variation of NC10 bacterial assemblages in the examined samples (*p* < 0.05, 1,000 Monte Carlo permutations; Fig. 4). In addition, Pearson moment correlation analyses showed that the number of OTUs was positively correlated with the ammonium content, TIN content and OrgC content (Table 2). The Chao1 estimator was found to be positively correlated with the ammonium content and TIN content (Table 2).

### Abundance of NC10 phylum bacteria and total bacteria

**Figure 5:**
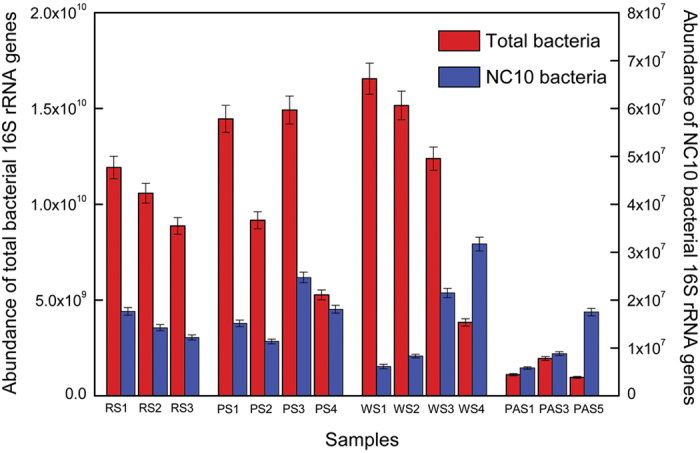
Copy numbers of the 16S rRNA genes of NC10 phylum bacteria and total bacteria in the examined freshwater habitats. Error bars indicate standard deviation (*n* = 3).

The abundance of NC10 bacteria in each sample was determined based on the quantification of their 16S rRNA genes, according to Ettwig *et al*.^13^. The qPCR results further confirmed the presence of NC10 bacteria in the all collected samples. The copy number of the 16S rRNA genes of NC10 bacteria ranged between 5.8 × 10^6^ and 3.2 × 10^7^ copies g^−1^ sediment/soil (Fig. 5). Pearson moment correlation analyses showed that no single environmental factor had significant correlation with the copy number of the 16S rRNA genes of NC10 bacteria. Furthermore, the copy number of the 16S rRNA genes of total bacteria was also determined in the examined samples, which varied from 9.7 × 10^8^ to 1.7 × 10^10^ copies g^−1^ sediment/soil (Fig. 5). According to the qPCR results, the percentages of NC10 bacteria in the examined habitats ranged from 0.06% to 1.81% (Fig. S3).

## Discussion

The present study provides a detailed comparison of NC10 bacterial communities in four different freshwater habitats (reservoir sediments, pond sediments, wetland sediments and paddy soils) based on Illumina-based 16S rRNA gene sequencing. Our study is the first comparison of community composition and diversity of NC10 bacteria in different freshwater habitats by using exactly the same experimental conditions. Illumina-based 16S rRNA gene sequencing and subsequent sequence analyses showed the presence of diverse NC10-related sequences in every examined sample. The results showed that the NC10 was the seventh to eleventh dominant phylum in the examined habitats. The percentages of NC10 bacteria of the total bacteria ranged from 0.4% to 2.5%, which were similar to the values reported for agricultural soils (0.8–4.5%)^30^. Thus the NC10 phylum could be an important part of the whole microbial community in freshwater habitats. Illumina-based 16S rRNA gene sequencing confirmed the co-existence of NC10 bacteria and anammox bacteria in each examined sediment/soil sample (Table S2). Both the NC10 bacteria and anammox bacteria use the nitrite as the electron acceptor, and they may compete for nitrite in nature as previously reported^10^. In the present study, the percentages of NC10 bacteria were higher than those of anammox bacteria in all examined samples (Table S2). However, there is no significant correlation (positive or negative) between the percentages of NC10 bacteria and the percentages of anammox bacteria in the examined samples. Thus the potential relationship between the NC10 bacteria and anammox bacteria needs to be further explored. Recently, Haroon *et al*.^43^ reported that the archaeon ‘*Candidatus* Methanoperedens nitroreducens’ (*M. nitroreducens*) can couple anaerobic oxidation of methane to nitrate reduction to nitrite. However, until now, the role of *M. nitroreducens*-like archaea in reducing methane emissions in natural habitats is nearly unknown. It is very possible that the *M. nitroreducens*-like archaea also exist in the examined habitats since nitrate is more abundant than nitrite in the examined sediments/soils (Table 1). It would be interesting to investigate the communities of NC10 bacteria, *M. nitroreducens*-like archaea and anammox bacteria together in future studies to reveal the potential relationship between the three different groups of microbes.

A high diversity of NC10 bacterial 16S rRNA genes was observed in the examined habitats, showing 40 to 97 OTUs in each habitat. In the first report of determining NC10 bacterial diversity using Illumina-based 16S rRNA gene sequencing, researchers also found a high NC10 bacterial 16S rRNA gene diversity in agricultural soils, with a total of 115 OTUs being observed^30^. Overall, the NC10 bacterial 16S rRNA gene diversity was obviously higher than those reported for freshwater habitats by using clone library analyses, like the reservoirs^44^,^45^,^46^, lakes^4^,^40^,^47^, rivers^17^, wetlands^7^,^9^,^10^,^18^ and paddy soils^8^,^19^,^20^. Therefore, the results of this study further confirm that the application of Illumina-based 16S rRNA gene sequencing using the less biased universal primers can help the detection of NC10 bacteria in environments. Among the examined habitats, the reservoir sediments showed the highest NC10 bacterial 16S rRNA gene diversity (Table 1). It can be observed that there are also some variations in the diversity of NC10 bacteria in samples from the same habitat (Table 1), suggesting that the local soil/sediment heterogeneity could provide different microenvironments for these bacteria.

With the help of the obtained high-quality 16S rRNA gene sequences, the present study determined the taxonomic assignment of the detected NC10 bacteria. The co-existence of different groups of NC10 bacteria was observed in each examined habitat, including group A, group B, group C and group D (Fig. 1), and group B members were the dominant NC10 bacteria (Fig. S2). Previous studies have also indicated the co-occurrence of different groups of NC10 bacteria in different natural environments^18^,^19^,^30^. Similarly, Shen *et al*.^30^ reported that the group B members were the dominant NC10 bacteria in different depths of agricultural soils by using Illumina-based 16S rRNA gene sequencing. Furthermore, it can be found that the microbial communities of NC10 bacteria from the four different habitats showed obvious differences (Fig. 3), suggesting that certain habitat types tend to contain unique NC10 bacterial communities. This also suggested that the habitat may influence the community structures of the NC10 bacteria in natural freshwater habitats. A Venn diagram showed that 30–63% of OTUs of NC10 bacteria were unique to a single sample. In contrast, less than 24% of OTUs were shared by either two or three habitats, and only 2% of OTUs co-occur in all four examined habitats (Fig. 3). These results also suggested that habitat can select for or influence NC10 bacterial community structures and may affect biogeography of NC10 bacteria. In addition, higher percentages of group A of NC10 bacteria, which were reported to be the dominant bacteria responsible for AMO coupled to nitrite reduction, were observed in reservoir sediments and pond sediments as compared with wetland sediments and paddy soils (Fig. S2). It is possible that the stable environmental conditions (e.g. stable anoxic condition) in relatively deep reservoir sediments and pond sediments seem to be beneficial for the slow-growing *M. oxyfera*^21^. It was reported that *M. oxyfera* can use a newly discovered intra-aerobic pathway for AMO coupled to nitrite reduction, producing oxygen without photosynthesis^14^,^48^. However, this microorganism was reported to be sensitive to oxygen^21^,^49^. It was observed that the group A members could only be detected in subsurface (20–30 cm) and deep (40–50 cm) paddy soils, while no sequences affiliated to group A could be recovered from surface (0–10 cm) soils (Fig. S2). This further suggested that the stable anoxic condition would be beneficial for the distribution of group A members. The relatively higher percentages of group A members observed in reservoir sediments and pond sediments indicated that AMO coupled to nitrite reduction may have a greater potential to play a role in methane oxidation in these two habitats. However, the information of AMO activity mediated by NC10 bacteria is not available, which should be assessed by the future stable isotope experiments.

The environmental factors could have important influence on the community composition and diversity of NC10 bacteria^22^. In the present study, CCA test showed that the TIN content and ammonium content were the two most important environmental factors influencing the community structures of NC10 bacteria in the examined freshwater habitats (Fig. 4). Pearson moment correlation analyses further suggested that the TIN content, ammonium content and OrgC content were important factors influencing the diversity of NC10 bacteria (Table 2), which are similar to previous findings in the Qiantang River sediments^17^, Mai Po wetland sediments^50^, Jiaojiang estuarine sediments^51^ and Yellow River estuarine sediments^52^. The ammonium content and OrgC content could influence the NC10 bacteria by influencing the production of nitrite and methane. The tight correlation between the ammonium content and the community structures of NC10 bacteria suggested a high possibility of the cooperation between NC10 bacteria and ammonium-oxidizing bacteria or archaea as previously indicated^17^,^46^. In addition, it should be noted that the *in situ* methane concentration that we did not measure in the present study could also be an important factor influencing the community structures of NC10 bacteria in the examined habitats. Therefore, the environmental factors that determine the community composition and diversity of NC10 bacteria in freshwater habitats are not comprehensively revealed because the limited factors were included in this study.

Quantitative PCR further confirmed the presence of NC10 bacteria in the examined sediments/soils, with the 16S rRNA gene copy numbers ranging from 5.8 × 10^6^ and 3.2 × 10^7^ copies g^−1^ sediment. These data are slightly higher than the ranges reported for lake sediments (10^5^–10^6^ copies g^−1^ sediment)^40^, but fall within the ranges reported for most river sediments (10^6^–10^7^ copies g^−1^ sediment)^17^, wetland sediments/soils (10^4^–10^7^ copies g^−1^ sediment/soil)^7^,^9^,^10^,^18^ and paddy soils (10^5^–10^8^ copies g^−1^ soil)^8^,^18^,^20^. Although a heterogeneous distribution of NC10 bacterial 16S rRNA gene abundance was found in the examined habitats (Fig. 5), no significant correlation was observed between NC10 bacterial 16S rRNA gene abundance and any single environmental factor measured in this study (Table 2). It is possible that an integrated effect of several factors rather than single one influences the abundance of NC10 bacteria in freshwater habitats as previously reported^18^.

## Conclusions

In this study, Illumina-based 16S rRNA gene sequencing using primers targeting universal bacterial 16S rRNA genes was used to describe and compare the community composition and diversity of NC10 phylum bacteria in four different freshwater habitats, including reservoir sediments, pond sediments, wetland sediments and paddy soils. A high diversity of NC10 bacterial 16S rRNA genes was observed in the examined habitats, ranging from 40 to 97 OTUs based on 3% sequence divergence. It was observed that the microbial communities of NC10 bacteria from the four different habitats showed obvious differences, suggesting that the habitat may influence the community structures of NC10 bacteria in natural freshwater habitats. In addition, the ammonium content and OrgC content were found to be the two most important environmental factors influencing the community structures of NC10 bacteria in the examined habitats. This study provides insights into the community structures and biogeography of NC10 bacterial communities in relationship to specific habitats.

## Additional Information

**How to cite this article**: Shen, L.-D. *et al*. Comparison of community structures of *Candidatus* Methylomirabilis oxyfera-like bacteria of NC10 phylum in different freshwater habitats. *Sci. Rep*. **6**, 25647; doi: 10.1038/srep25647 (2016).

## Supplementary Material

Supplementary Information

## Figures and Tables

**Table 1:** Diversity of the 16S rRNA genes of NC10 phylum bacteria in different freshwater habitats.

Freshwater habitats	No. of sequences	Coverage (%)	No. of OTUs	Shannon index	Chao1 estimator
**RS**	**1128**	**98.0**	**97**	**3.09**	**104.2**
RS1	261	90.4	45	2.43	82.5
RS2	217	90.3	44	2.73	65.0
RS3	650	95.8	75	2.53	89.0
**PS**	**981**	**99.6**	**46**	**2.65**	**46.2**
PS1	358	99.7	20	2.10	20.0
PS2	170	97.1	19	2.42	21.5
PS3	94	91.5	19	2.44	47.0
PS4	359	98.9	27	2.61	28.2
**WS**	**4811**	**99.8**	**61**	**2.00**	**63.8**
WS1	1054	98.7	38	2.00	48.1
WS2	729	98.9	27	1.88	31.7
WS3	2002	99.3	44	1.81	57.1
WS4	1026	98.5	42	2.10	59.5
**PAS**	**992**	**99.1**	**40**	**2.64**	**44.5**
PAS10	28	92.9	11	1.70	12.0
PAS30	580	99.0	33	2.53	35.1
PAS50	384	98.7	26	2.54	29.3

RS—reservoir sediments; PS—pond sediments; WS—wetland sediments; PAS—paddy soils; PAS10—upper 0–10 cm soil; PAS30—20–30 cm soil; PAS50—40–50 cm soil.

**Table 2:** Pearson moment correlation analyses of environmental factors and NC10 phylum bacterial diversity and abundance.

Environmental factors	Pearson correlation coefficient			
	No. of OTUs	Shannon index	Chao1	Abundance
pH	−0.066	0.2007	−0.082	0.126
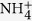 -N	**0.278**	−0.075	**0.317**	−0.082
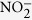 -N	0.089	−0.024	0.031	−0.077
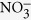 -N	0.094	−0.006	0.185	−0.081
TIN	**0.294**	−0.083	**0.359**	−0.083
OrgC	**0.231**	−0.033	0.124	−0.030

TIN—total inorganic nitrogen; OrgC—organic carbon; Boldface denotes a *p* value of <0.05, which is typically regarded as significant, as determined by SPSS version 15.0 program (SPSS, Chicago, Illinois, USA).
