# Supplementary material for: Comparison of community structures of Candidatus Methylomirabilis oxyfera-like bacteria of NC10 phylum in different freshwater habitats
Source: Sci Rep. 2016 May 9;6:25647. doi: 10.1038/srep25647 (PMC4860643; doi:10.1038/srep25647)
Supplement: Supplementary Information [file srep25647-s1.pdf]

Supporting Information

Comparison of community structures of *Candidatus* Methylomirabilis oxyfera-like bacteria of NC10 phylum in different freshwater habitats

Li-dong Shen[1,2*], Hong-sheng Wu[1,2], Zhi-qiu Gao[3,4], Xu Liu[2], Ji Li[2]

[1]Collaborative Innovation Center on Forecast and Evaluation of Meteorological Disasters, Jiangsu Key Laboratory of Agricultural Meteorology, College of Applied Meteorology, Nanjing University of Information Science and Technology, Nanjing, 210044, China

[2]Department of Agricultural Resource and Environment, College of Applied Meteorology, Nanjing University of Information Science and Technology, Nanjing 210044, China

[3]State Key Laboratory of Atmospheric Boundary Layer Physics and Atmospheric Chemistry, Institute of Atmospheric Physics, Chinese Academy of Science, Beijing, China

[4]College of Geophysics and Remote Sensing, Nanjing University of Information Science and Technology, Nanjing 210044, China

***For correspondence**

Dr. Li-dong Shen

Collaborative Innovation Center on Forecast and Evaluation of Meteorological Disasters, Jiangsu Key Laboratory of Agricultural Meteorology, College of Applied Meteorology, Nanjing University of Information Science and Technology, Nanjing, 210044, China

E-mail: shenld@nuist.edu.cn

Table S1 Basic physiochemical properties of the examined freshwater habitats

Table S2 Percentages of NC10 phylum sequences, Planctomycetes phylum sequences and anammox-related sequences in the examined sediment/soil samples

Fig. S1 Melting curve analyses of the qPCR products of NC10 phylum bacteria (a) and total bacteria (b) from the examined freshwater habitats

Fig. S2 Community composition of NC10 phylum bacteria in different samples collected from the examined freshwater habitats

Fig. S3 Percentages of NC10 phylum bacteria in different samples collected from the examined freshwater habitats based on qPCR results

Table S1 Basic physiochemical properties of the examined freshwater habitats

Sediment/soil samples	pH	NH_4^+-N (mg kg^{-1})	NO_2^--N (mg kg^{-1})	NO_3^--N (mg kg^{-1})	TIN (mg kg^{-1})	OrgC (g kg^{-1})
**RS**						
RS1	7.3	4.5	0.1	0.8	5.4	13.1
RS2	7.1	3.2	0.1	1.3	4.6	12.9
RS3	6.9	5.3	0.2	1.4	6.9	12.5
**PS**						
PS1	7.3	52.5	0.2	28.2	80.9	29.7
PS2	7.5	174.6	0.4	15.3	190.3	27.1
PS3	7.2	75.3	0.5	16.2	92.0	30.4
PS4	7.6	115.2	0.2	49.2	164.6	25.6
**WS**						
WS1	6.2	43.1	0.1	29.1	72.5	12.8
WS2	6.5	20.8	0.2	15.6	36.6	10.5
WS3	6.9	38.9	0.1	19.4	58.4	11.2
WS4	7.1	30.1	0.1	17.6	47.8	14.6
**PAS**						
PAS10	6.7	56.9	0.2	17.8	74.9	15.2
PAS30	7.1	45.1	0.4	8.7	54.2	13.9
PAS50	6.8	36.9	0.1	3.3	40.3	13.1

RS—reservoir sediments; PS—pond sediments; WS—wetland sediments; PAS—paddy soils; PAS10—upper 0-10 cm soil; PAS30—20-30 cm soil; PAS50—40-50 cm soil; TIN—total inorganic nitrogen; OrgC—organic carbon

Table S2 Percentages of NC10 phylum sequences, Planctomycetes phylum sequences and anammox-related sequences in the examined sediment/soil samples

Sediment/soil samples	NC10 phylum sequences	Planctomycetes phylum sequences	Anammox-related sequences
RS1	1.3%	0.6%	0.081%
RS2	1.5%	0.6%	0.079%
RS3	1.0%	0.4%	0.045%
PS1	0.6%	1.0%	0.055%
PS2	0.3%	0.9%	0.065%
PS3	0.1%	0.2%	0.036%
PS4	0.7%	0.7%	0.040%
WS1	1.6%	1.6%	0.108%
WS2	1.9%	2.5%	0.081%
WS3	4.5%	0.8%	0.065%
WS4	2.6%	2.0%	0.112%
PAS10	0.1%	0.3%	0.041%
PAS30	1.7%	0.4%	0.031%
PAS50	2.0%	0.1%	0.012%

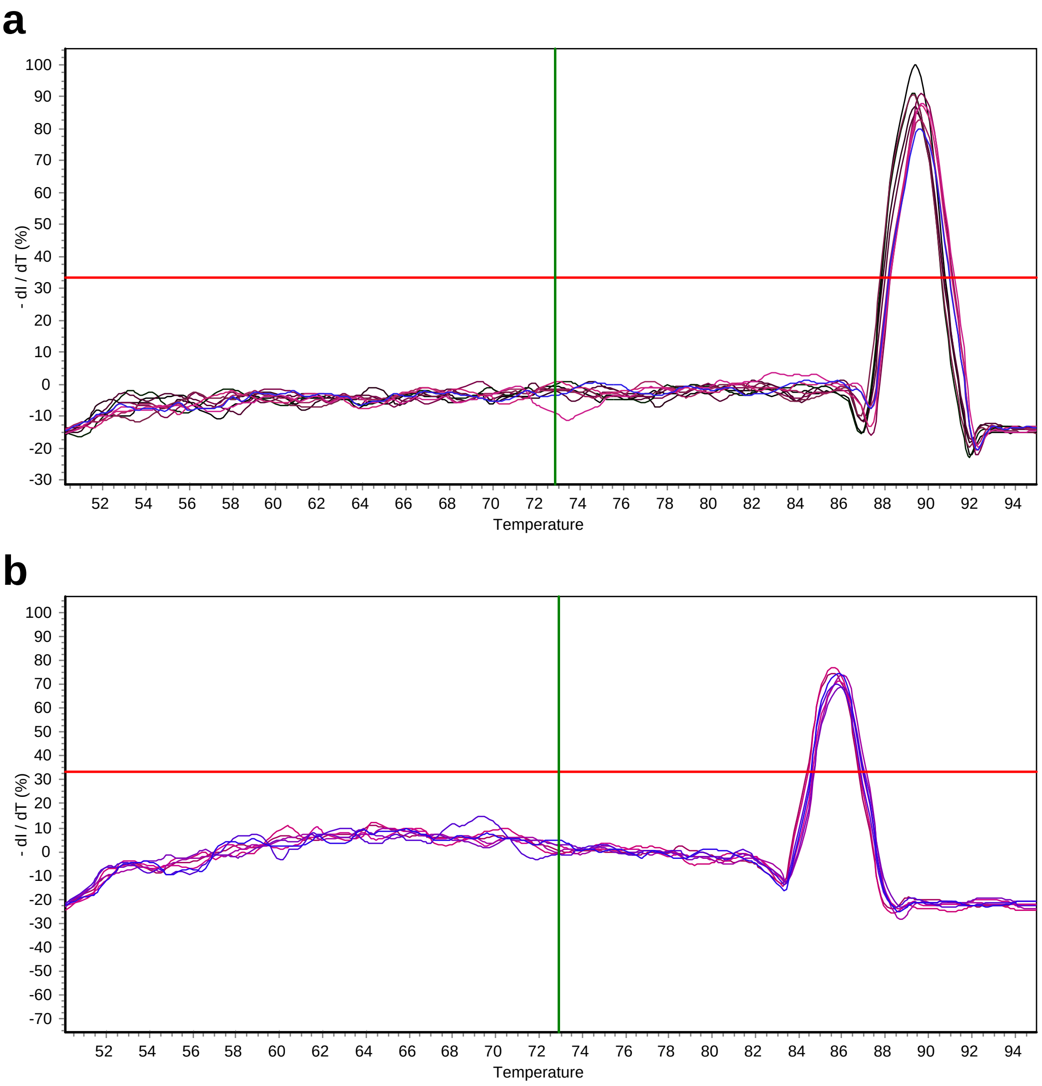

Fig. S1

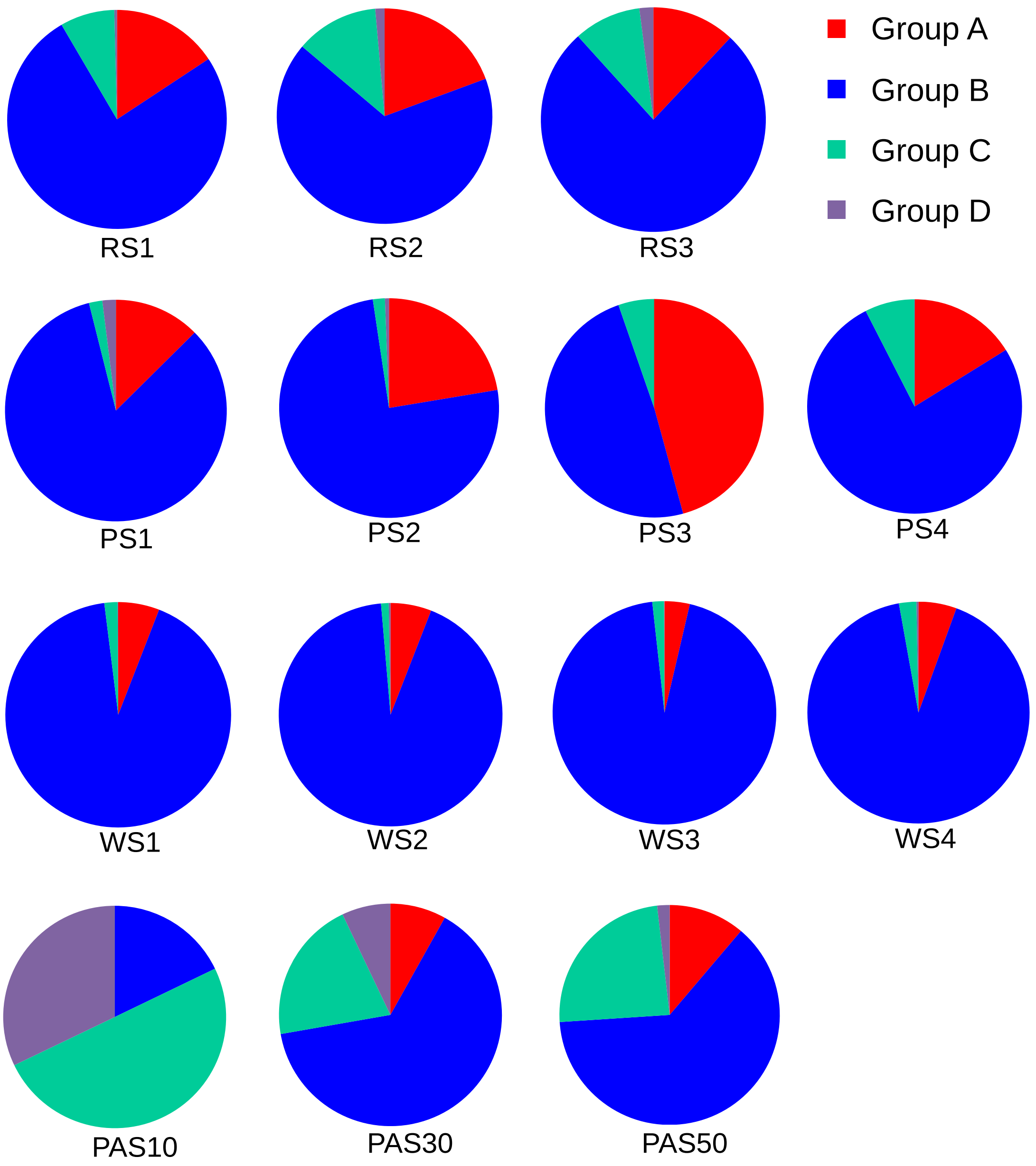

Group A
Group B
Group C
Group D
RS1
RS2
RS3
PS1
PS2
PS3
PS4
WS1
WS2
WS3
WS4
PAS10
PAS30
PAS50

Fig. S2

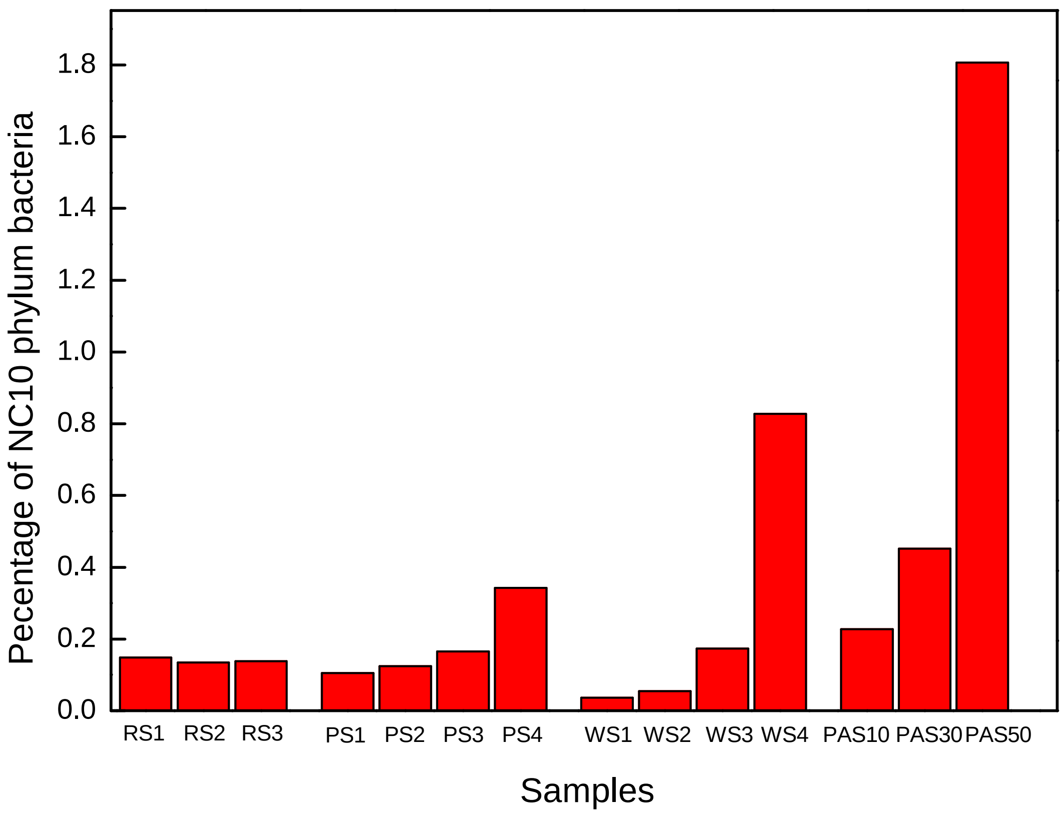
Pecentage of NC10 phylum bacteria
0.0
0.2
0.4
0.6
0.8
1.0
1.2
1.4
1.6
1.8
RS1
RS2
RS3
PS1
PS2
PS3
PS4
WS1
WS2
WS3
WS4
PAS10
PAS30
PAS50
Samples

Fig. S3
